# Near-Field Three-Dimensional Planar Millimeter-Wave Holographic Imaging by Using Frequency Scaling Algorithm

**DOI:** 10.3390/s17102438

**Published:** 2017-10-24

**Authors:** Ye Zhang, Bin Deng, Qi Yang, Jingkun Gao, Yuliang Qin, Hongqiang Wang

**Affiliations:** College of Electronic Science, National University of Defense Technology, Changsha 410073, China; fighting_zy10@126.com (Y.Z.); yangqi_nudt@163.com (Q.Y.); oscar92923@163.com (J.G.) qinyuliang@nudt.edu.cn (Y.Q.), oliverwhq@tom.com (H.W.)

**Keywords:** frequency scaling algorithm, near-field, millimeter-wave, 3-D holographic imaging

## Abstract

In this paper, a fast three-dimensional (3-D) frequency scaling algorithm (FSA) with large depth of focus is presented for near-field planar millimeter-wave (MMW) holographic imaging. Considering the cross-range range coupling term which is neglected in the conventional range migration algorithm (RMA), we propose an algorithm performing the range cell migration correction for de-chirped signals without interpolation by using a 3-D frequency scaling operation. First, to deal with the cross-range range coupling term, a 3-D frequency scaling operator is derived to eliminate the space variation of range cell migration. Then, a range migration correction factor is performed to compensate for the residual range cell migration. Finally, the imaging results are obtained by matched filtering in the cross-range direction. Compared with the conventional RMA, the proposed algorithm is comparable in accuracy but more efficient by using only chirp multiplications and fast Fourier transforms (FFTs). The algorithm has been tested with satisfying results by both simulation and experiment.

## 1. Introduction

The millimeter-wave (MMW) imaging technique holds large potential in the application of security inspection for its unique electromagnetic properties [1,2]. Unlike optical and infrared radiation, MMW offers the property of being able to ‘‘see through’’ non-polar and non-metallic materials such as clothing, plastic, and cardboard with relatively little energy loss. Compared to microwaves and radio-frequency waves, MMW can achieve better spatial resolution due to its shorter wavelength and make concealed weapons easier to identify. Moreover, unlike the X-ray backscatter imaging technique, MMW is harmless to human beings and is more likely to be accepted. Therefore, it is suitable for detecting concealed threats in airport, stations, and other public places.

In recent years, many facilities have done research on security inspection using MMW imaging systems, such as the Pacific Northwest National Laboratory (PNNL) [3,4,5], Rohde Schwarz Company [6,7,8], Tsinghua University [9], and so on [10,11]. The MMW imaging system transmits wideband electromagnetic waves with a spherical wave front to illuminate objects and reconstruct three-dimensional (3-D) images using the amplitude and phase of the recorded reflected signals. The system structure mainly includes a two-dimensional (2-D) uniform planar synthetic aperture [3,9], a cylindrical synthetic aperture [4], and a 2-D sparse planar synthetic aperture [6,7,8]. In this paper, we concentrate on the 2-D uniform planar synthetic aperture to achieve 3-D image reconstruction, which is considered as holographic radar imaging and also can be regarded as 3-D synthetic aperture radar imaging. The 2-D uniform planar synthetic aperture is usually formed by electrical scanning in the horizontal direction and mechanical scanning in the vertical direction with a linear antenna array.

For near-field wideband planar MMW holographic imaging, linear frequency modulation continuous wave (LFMCW) and stepped frequency continuous wave (SFCW) are the most popular signal systems because of the large time bandwidth product which brings high gain and high resolution together. A series of algorithms have been developed, such as time-domain correlation algorithm (TDCA), back projection algorithm (BPA) [12], range migration algorithm (RMA) [3,9], and range stacking algorithm (RSA) [13]. Considering the application background of safety inspection, the target distance is usually within one meter, but the distance is not known exactly, therefore, an imaging algorithm with a large depth of focus is required. The TDCA and BPA are time-domain algorithms that can achieve accurate imaging by coherent accumulation of each point in the imaging scene, however, the amount of computation is proportional to the number of points leading to a significant computation time which limits the real-time applications. RMA is an accurate algorithm with excellent precision, and it adopts interpolation in a spatial domain to eliminate the spherical curve, which leads to space variation of range cell migration and range cell migration in the 3-D spatial spectrum. Until now, most near-field wideband planar MMW holographic imaging systems adopted RMA as the imaging algorithm, and a series of improvements have been made based on RMA such as replacing interpolation and fast Fourier transform (FFT) by non-uniform FFT [14], compensating for the antenna position error which is caused by mechanical scanning of linear antenna array [15], and so on. However, the interpolation process implies a high computational cost which slows down the imaging speed, and the image reconstruction accuracy is also limited by interpolating kernel function and the number of interpolation points. The RSA is a completely accurate algorithm without interpolation which adopts different compensating distances to eliminate the spherical curve in the corresponding distance and composes corresponding imaging results into the final 3-D reconstruction image, but the computational complexity is far greater than that of RMA if the target is thick in the range direction. Concerning synthetic aperture radar (SAR) processing, there are a family of frequency domain approximation algorithms such as range-Doppler algorithm (RDA) [16], chirp scaling algorithm (CSA) [17], and frequency scaling algorithm (FSA) [18]. Gimeno [19] has extended the original 2-D CSA to 3-D near-field wideband radar imaging, but the CSA cannot be applied directly on the de-chirped signal. Ge Jiang [20] has extended RDA to 3-D near-field wideband radar imaging, but the focusing depth is limited. So far, there is no literature applying FSA to near-field wideband planar MMW holographic imaging.

In this paper, we present a fast three-dimensional (3-D) frequency scaling algorithm with large depth of focus for near-field planar millimeter-wave holographic imaging. The proposed algorithm performs range cell migration correction for de-chirped signals based on LFMCW without interpolation by using only chirp multiplications and FFTs. Compared with the conventional RMA, the proposed algorithm is comparable in accuracy but more efficient.

In the next section, the near-field planar millimeter-wave holographic imaging scene and the proposed fast three-dimensional (3-D) frequency scaling algorithm with large depth of focus is described. In Section 3, both point targets simulation results and experimental results in the 35 GHz band are performed to verify the effectiveness of the algorithm. Finally, Section 4 summarizes this paper.

## 2. Model and Method

**Figure 1:**
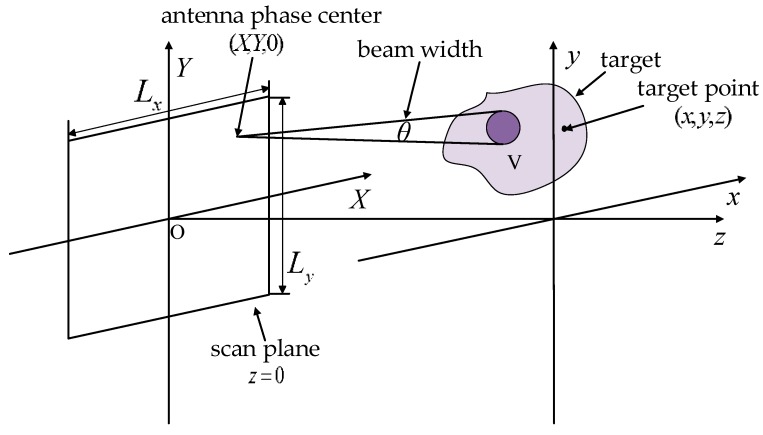
Geometry of near-field planar millimeter-wave holographic imaging.

Figure 1 illustrates a typical geometry of near-field planar millimeter-wave holographic imaging. An ideal point target is located at position (x,y,z) with scattering intensity σ in the Cartesian coordinate system. The signal is transmitted and received by a pair of antennas whose equivalent phase center is located at capital coordinate (X,Y,0) and the quasi monostatic planar antenna array is formed by mechanical scanning in the vertical direction with a linear antenna array. The antenna transmits a linear frequency modulation continuous wave with large time bandwidth product and receives the echo signal in a de-chirping manner with a reference distance $R_{ref}$. Ignoring the energy loss during the transmission process and assuming the 3-D envelope of the echo signal is rectangular, then the echoed data of the ideal point target takes the form of

(1) $$\begin{array}{cc} s\;(X,Y,t) & =\sigma \mathrm{rect}\;(\frac{X}{L_{X}})\;\mathrm{rect}\;(\frac{Y}{L_{Y}})\;\mathrm{rect}\;(\frac{t-2R/c}{T_{p}})\\ & \times \exp\;(-j\frac{4\pi }{c}(\gamma (t-\frac{2R_{ref}}{c})+f_{c})\;(R-R_{ref}))\;\exp\;(j\frac{4\pi \gamma }{c^{2}}{\left(R-R_{ref}\right)}^{2}) \end{array}$$

where

(2) $$R=\sqrt{{\left(X-x\right)}^{2}+{\left(Y-y\right)}^{2}+z^{2}}$$

Here, R is the distance between target and antenna probe, c is the speed of light, $f_{c}$ is center frequency, γ is the chirp rate of LFMCW, $T_{p}$ is the time length of signal, $L_{X}$ and $L_{Y}$ are the length and height of the planar antenna array, respectively.

The second exponential term in Equation (1) is residual video phase (RVP) which is introduced by a de-chirping manner and should be removed by phase compensation in most imaging algorithms. However, in this paper, the 3-D frequency scaling algorithm is realized based on the RVP. For the convenience of the latter discussion, the waveform is moved forward by $2R_{ref}/c$. On the basis of the stationary phase principle, the Equation (1) can be transformed as

(3) $$s\;(X,Y,t)=A\;(X,Y,t)\;\exp\;(-j\frac{4\pi }{c}(\gamma t+f_{c})\;(R-R_{ref}))⊗\exp\;(-j\pi \gamma t^{2})$$

where $A\;(X,Y,t)=\sigma \mathrm{rect}\;(\frac{X}{L_{X}})\;\mathrm{rect}\;(\frac{Y}{L_{Y}})\;\mathrm{rect}\;(\frac{t-2R/c}{T_{p}})$ is the product of target scattering intensity and the 3-D envelope of the echo signal in order to simplify the formula expression, and symbol ⊗ indicates convolution. In order to prove the equivalence of Equations (1) and (3), the theory of stationary phase principle should be illustrated. The stationary phase principle is used for the integration of oscillatory signals with slowly varied amplitude. Except for the zero frequency and its adjacent area, the rest of the integral signal changes rapidly between positive and negative and has no contribution to integral result. The echo signal in Equation (1) approximates to a single frequency signal with frequency $-2(R-R_{ref})/c$, and the echo envelope is also proportional to the time delay $2\;(R-R_{ref})/c$. According to the theory of stationary phase principle, only the area with frequency $-2\;(R-R_{ref})/c$ of the chirp signal $\exp\;(-j\pi \gamma t^{2})$ makes contribution to the integral result in Equation (3) and then it can be transformed to Equation (1) by numerical integration. The detailed formula deduction process is given in the appendix of [18].

Let $K_{Rc}=4\pi f_{c}/c$, $\Delta K_{R}=4\pi \gamma t/c$, $K_{R}=K_{Rc}+\Delta K_{R}$, we have

(4) $$s\;(X,Y,\Delta K_{R})=A\;(X,Y,\Delta K_{R})\;\exp\;(-jK_{R}(R-R_{ref}))⊗\exp\;(-j\frac{\Delta K_{R}^{2}}{2b})$$

where $b=8\pi \gamma /c^{2}$. In order to eliminate the spherical curve which leads to space variation of range cell migration and range cell migration in 3-D spatial spectrum, the echo signal needs to be converted to the spatial spectrum domain. After performing the 2-D spatial Fourier transformation of the echo signal $s\;(X,Y,\Delta K_{R})$ over variables X and Y, we can obtain the spatial spectrum based on the stationary phase principle

(5) $$\begin{array}{cc} S\;(K_{x},K_{y},\Delta K_{R}) & =A\;(K_{x},K_{y},\Delta K_{R})\;\exp\;(jK_{R}R_{ref})\;\exp\;(-j\sqrt{K_{R}^{2}-K_{x}^{2}-K_{y}^{2}}z)\\ & \times \exp\;(-jK_{x}x)\;\exp\;(-jK_{y}y)⊗\exp\;(-j\frac{\Delta K_{R}^{2}}{2b}) \end{array}$$

where $K_{x}$ and $K_{y}$ indicate the spatial frequency corresponding to variables X and Y, respectively. Taking the cross-range range coupling term $\exp\;(-j\sqrt{K_{R}^{2}-K_{x}^{2}-K_{y}^{2}}z)$ into consideration, the Equation (5) can be transformed as follows based on the Taylor expansion over $\Delta K_{R}$.

(6) $$\begin{array}{cc} S(K_{x},K_{y},\Delta K_{R}) & =A_{1}(K_{x},K_{y},\Delta K_{R})\;\exp\;(-j(\frac{z}{A_{XY}}-R_{ref})\Delta K_{R})\;\exp\;(-jA_{XY}K_{Rc}z-jK_{x}x-jK_{y}y)\\ & \times \exp\;(j\frac{(K_{x}^{2}+K_{y}^{2})z}{2K_{Rc}^{3}A_{XY}^{3}}\Delta K_{R}^{2})\;\exp\;(-j\frac{(K_{x}^{2}+K_{y}^{2})z}{2K_{Rc}^{4}A_{XY}^{5}}\Delta K_{R}^{3})⊗\exp\;(-j\frac{\Delta K_{R}^{2}}{2b}) \end{array}$$

where

(7) $$A_{XY}=\sqrt{1-\frac{K_{x}^{2}+K_{y}^{2}}{K_{Rc}^{2}}}$$

(8) $$A_{1}\;(K_{x},K_{y},\Delta K_{R})=A\;(K_{x},K_{y},\Delta K_{R})\;\exp\;(jK_{Rc}R_{ref})$$

In order to simplify the formula expression, the Taylor expansion is limited to the third-order. From Equation (6), we can find that the scaling factor of vertical distance z is $1/A_{XY}$ and it varies with $K_{x}$ and $K_{y}$. In order to eliminate the space variation of range cell migration, a 3-D frequency scaling function is introduced

(9) $$H_{FS}(K_{x},K_{y},\Delta K_{R})=\exp\;(-j\frac{\Delta K_{R}^{2}}{2b}(A_{XY}-1))$$

Multiply Equation (9) with Equation (6) and we can obtain

(10) $$\begin{array}{cc} S_{1}\;(K_{x},K_{y},\Delta K_{R}) & =A_{2}\exp\;(-j(z-A_{XY}R_{ref})\;\Delta K_{R})\;\exp\;(-jA_{XY}K_{Rc}z-jK_{x}x-jK_{y}y)\\ & \times \exp\;(j\frac{(K_{x}^{2}+K_{y}^{2})z}{2K_{Rc}^{3}A_{XY}}\Delta K_{R}^{2})\;\exp\;(-j\frac{(K_{x}^{2}+K_{y}^{2})z}{2K_{Rc}^{4}A_{XY}^{2}}\Delta K_{R}^{3})\\ & \times \exp\;(-j\frac{A_{XY}\Delta K_{R}^{2}}{2b}(A_{XY}-1))⊗\exp\;(-j\frac{A_{XY}\Delta K_{R}^{2}}{2b}) \end{array}$$

The derivation is given in the Appendix. It can be seen from Equation (10) that the scaling factor of vertical distance z is constant and the space variation of range cell migration has been eliminated. The RVP will be removed in the following step based on the FFT to achieve de-convolution, and we can obtain

(11) $$\begin{array}{cc} S_{2}(K_{x},K_{y},\Delta K_{R}) & ={\mathrm{FT}}_{\Delta K_{R}}({\mathrm{IFT}}_{\Delta K_{R}}(S_{1}\;(K_{x},K_{y},\Delta K_{R}))\cdot \mathrm{conj}\;({\mathrm{IFT}}_{\Delta K_{R}}(\exp\;(-j\frac{A_{XY}\Delta K_{R}^{2}}{2b}))))\\ & =A_{2}\exp\;(-j\;(z-A_{XY}R_{ref})\;\Delta K_{R})\;\exp\;(-jA_{XY}K_{Rc}z-jK_{x}x-jK_{y}y)\\ & \times \exp\;(j\frac{(K_{x}^{2}+K_{y}^{2})z}{2K_{Rc}^{3}A_{XY}}\Delta K_{R}^{2})\;\exp\;(-j\frac{(K_{x}^{2}+K_{y}^{2})z}{2K_{Rc}^{4}A_{XY}^{2}}\Delta K_{R}^{3})\;\exp\;(-j\frac{A_{XY}\Delta K_{R}^{2}}{2b}(A_{XY}-1)) \end{array}$$

where conj ( ) indicates conjugate function, ${\mathrm{IFT}}_{\Delta K_{R}}$ indicates the inverse Fourier transform over $\Delta K_{R}$, and ${\mathrm{FT}}_{\Delta K_{R}}$ indicates the corresponding Fourier transform. The last exponential term in Equation (11) is the quadratic phase error introduced by $H_{FS}$, and it can be eliminated by multiplying the inverse frequency scaling function

(12) $$H_{IFS}\;(K_{x},K_{y},\Delta K_{R})=\exp\;(j\frac{A_{XY}\Delta K_{R}^{2}}{2b}\;(A_{XY}-1))$$

Then we can obtain

(13) $$\begin{array}{cc} S_{3}(K_{x},K_{y},\Delta K_{R}) & =A_{2}\exp\;(-j\;(z-A_{XY}R_{ref})\Delta K_{R})\;\exp\;(-jA_{XY}K_{Rc}z-jK_{x}x-jK_{y}y)\\ & \times \exp\;(j\frac{(K_{x}^{2}+K_{y}^{2})z}{2K_{Rc}^{3}A_{XY}}\Delta K_{R}^{2})\;\exp\;(-j\frac{(K_{x}^{2}+K_{y}^{2})z}{2K_{Rc}^{4}A_{XY}^{2}}\Delta K_{R}^{3}) \end{array}$$

So far, the scaling operation has been accomplished, and the range curve of targets located at different distances is the same. Therefore, a linear phase function can be multiplied in the 3-D spatial frequency domain to compensate for the stationary range migration. In addition, the high order exponential term of $\Delta K_{R}$ should be compensated by secondary range compression. The range migration correction function (RMCF) and secondary range compressing function (SRCF) are

(14) $$H_{RMC}\;(K_{x},K_{y},\Delta K_{R})=\exp\;(-j\;(A_{XY}R_{ref}-z_{c})\;\Delta K_{R})$$

(15) $$H_{SRC}\;(K_{x},K_{y},\Delta K_{R})=\exp\;(-j\frac{(K_{x}^{2}+K_{y}^{2})\;z}{2K_{Rc}^{3}A_{XY}}\Delta K_{R}^{2})\;\exp\;(j\frac{(K_{x}^{2}+K_{y}^{2})\;z}{2K_{Rc}^{4}A_{XY}^{2}}\Delta K_{R}^{3})$$

The SRCF has spatial varying distortion and we can replace z with the center distance $z_{c}$ of the imaging scene to compensate for it. It should be noted that the step of secondary range compressing can be neglected here. Different from the imaging scene in SAR, the target distance z in MMW holographic imaging is always within one meter, and the phase value in exponential terms of SRCF is quite small that nearly has no impact on the final imaging results. This is also the reason why the proposed FSA has a large depth of focus. After range migration correction and secondary range compressing, we can obtain

(16) $$S_{4}(K_{x},K_{y},\Delta K_{R})=A_{2}\;\exp\;(-j\Delta K_{R}(z-z_{c}))\;\exp\;(-jA_{XY}K_{Rc}z-jK_{x}x-jK_{y}y)$$

**Figure 2:**
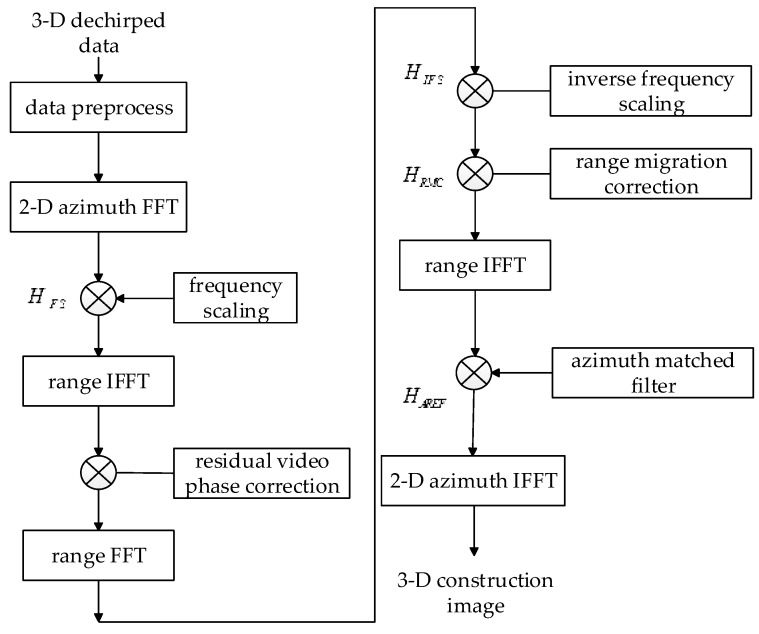
Flow chart of the near-field wideband 3-D frequency scaling algorithm. FFT = fast Fourier transform, IFFT = inverse fast Fourier transform, $H_{FS}$ = frequency scaling function, $H_{IFS}$ = inverse frequency scaling function, $H_{RMC}$ = range migration correction function, $H_{AREF}$ = azimuth reference function.

Implementing inverse fast Fourier transforms (IFFT) over $\Delta K_{R}$ in Equation (16) to achieve range compression, we can obtain

(17) $$S_{5}\;(K_{x},K_{y},Z)=A_{3}\mathrm{sinc}\;(\frac{bcT_{P}}{4}(Z+z_{c}-z))\;\exp\;(-jA_{XY}K_{Rc}z-jK_{x}x-jK_{y}y)$$

where Z indicates the spatial domain corresponding to variable $\Delta K_{R}$. By multiplying Equation (16) by the azimuth reference function $H_{AREF}(K_{x},K_{y},z)=\exp\;(jA_{XY}K_{Rc}z)$ and then implementing 2-D IFFT over the $K_{x}$ and $K_{y}$ dimensions, we can get the final imaging result of the targets to be imaged. To summarize, the flow chart of the proposed algorithm is displayed in Figure 2.

## 3. Results and Analysis

### 3.1. Computational Complexity

In order to evaluate the computational cost of the 3-D FSA, the echoed data are assumed to be recorded at $N_{x}\times N_{y}$ positions in the x×y plane with $N_{f}$ sampling points in the frequency domain. The computational cost depends on the total number of real multiplications, real additions, and sine or cosine calculations, as given in Table 1.

The conventional holographic imaging method is RMA and the 3-D image is reconstructed by

(18) $$\sigma \;(x,y,z)={\mathrm{IFT}}_{k_{x},k_{y},k_{z}}({\mathrm{STOLT}}_{k_{z}}({\mathrm{FT}}_{X,Y}\;(s(X,Y,t))\;\exp\;(jk_{z}z_{c})))$$

where ${\mathrm{STOLT}}_{k_{z}}$ indicates Stolt interpolation over $k_{z}$ since the spatial frequency is non-uniformly distributed, ${\mathrm{FT}}_{X,Y}$ indicates the 2-D Fourier transform from (X,Y) to $\left(k_{x},k_{y}\right)$, ${\mathrm{IFT}}_{k_{x},k_{y},k_{z}}$ indicates the 3-D inverse Fourier transform from $\left(k_{x},k_{y},k_{z}\right)$ to (x,y,z), $z_{c}$ is the center distance of the imaging scene, σ(x,y,z) is scattering intensity of the target, and s(X,Y,t) is the echo data. The computational complexity of RMA is mainly limited by the interpolating kernel function and the number of interpolation points, and the interpolation process implies a far higher computational cost than the other steps. Compared with 3-D RMA, the proposed 3-D FSA uses only chirp multiplications and FFTs which have higher efficiency.

### 3.2. Point Targets Simulations

To demonstrate the effectiveness of the 3-D FSA proposed in the previous section, a simulation with point targets was performed based on the near-field planar millimeter-wave holographic imaging model. The center frequency of the transmitted LFMCW is set to 35 GHz and the bandwidth is set to 5 GHz with 625 steps. The beam width is set to 45 degrees in both *x*- and *y*-directions. In order to satisfy the Nyquist sampling theorem to avoid aliasing in azimuth, the antenna scans along a planar array of 64 cm × 64 cm with a sample interval of 5 mm. There are five ideal point targets in the image area with unit scatter intensity and the coordinates of the point targets are shown in Table 2.

**Figure 3:**
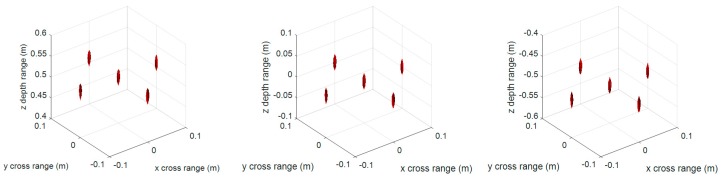
3-D imaging results of the conventional range migration algorithm (RMA) with compensation distance 0 (**left**), 0.51 m (**middle**), and 1.02 m (**right**).

**Figure 4:**
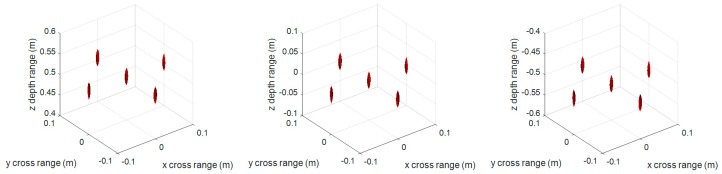
3-D imaging results of the proposed frequency scaling algorithm (FSA) with compensation distance 0 (**left**), 0.51 m (**middle**), and 1.02 m (**right**).

Figure 3 and Figure 4 are the 3-D reconstructed images by compensating different center distances $z_{c}$ of the imaging scene with conventional RMA and proposed FSA, respectively. The dynamic range in both Figure 3 and Figure 4 is −20 ~ 0 dB. As can be seen, the different compensation distance has almost no effect on the 3-D imaging results of RMA and FSA. It indicates that the proposed 3-D FSA also has a large depth of focus.

**Figure 5:**
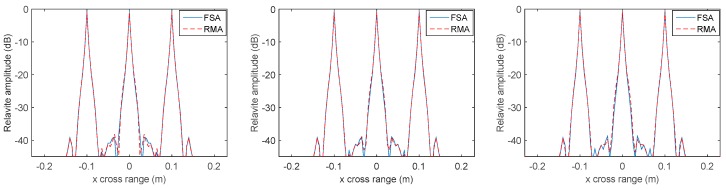
Profile comparisons along *x* with compensating distance 0 (**left**), 0.51 m (**middle**), and 1.02 m (**right**) by RMA and FSA.

**Figure 6:**
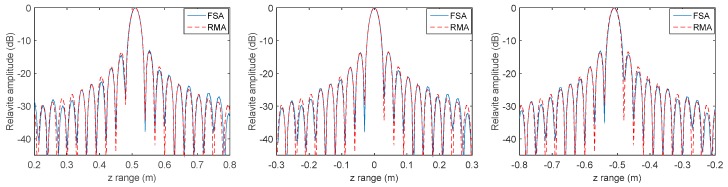
Profile comparisons along *z* with compensating distance 0 (**left**), 0.51 m (**middle**), and 1.02 m (**right**) by RMA and FSA.

MMW holographic imaging is concerned with azimuth resolution and range resolution so as to identify the target. The azimuth resolution of the proposed FSA should be nearly the same as RMA if the space variation of range cell migration and range cell migration have already been removed. As we know, the azimuth resolution will reduce if there still exists residual range migration. Taking these conditions into consideration, we concentrated on the comparison of azimuth profile and range profile to evaluate the effectiveness of the proposed FSA. It should be noted that the azimuth profile along y is the same as along x and can be neglected. Figure 5 shows the profile along x in the targets plain by compensating different center distances of the imaging scene with conventional RMA and proposed FSA. Figure 6 shows the profile along z of the point target located at (0, 0, 0.51) by compensating different center distances of the imaging scene with conventional RMA and proposed FSA. Here, there are eight times the data interpolation in the range profile and no interpolation in azimuth profile. As is known, RMA is an accurate algorithm with excellent precision and the compensation distance has no effect on its performance in range resolution and azimuth resolution. We can find that the azimuth profile and range profile is nearly the same in every compensating distance of the two methods, and it illustrates that the proposed FSA is comparable in accuracy with conventional RMA.

All the results of this paper were obtained on a laptop with Intel core i7-6500U 2.50 GHz central processing unit (CPU) and 8 GB random access memory (RAM) using Matlab codes. In order to guarantee the imaging quality of RMA, the interpolation interval was chosen as the spatial frequency sampling interval of the transmitted signal and the interpolation kernel function was the interp1 function in Matlab with linear mode. The computational time of RMA and FSA were 15.2109 s and 5.4883 s, respectively. Compared with the conventional RMA, the proposed algorithm is more efficient.

**Figure 7:**
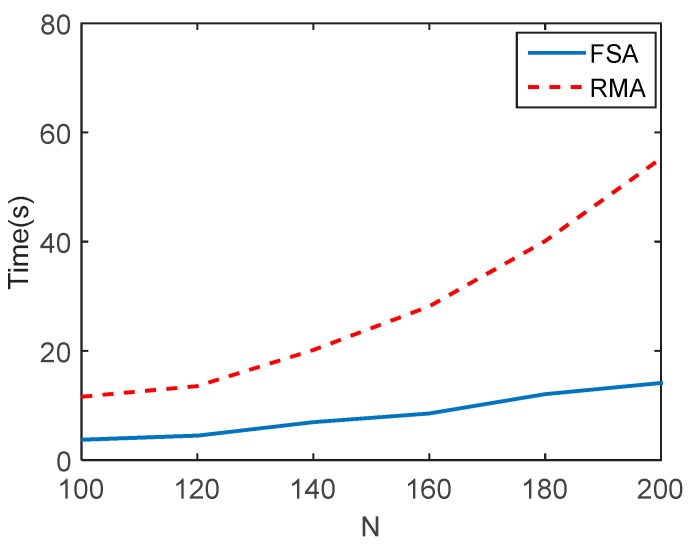
The relationship between imaging time and the number of array elements.

To further compare the efficiency of RMA and FSA, the relationship between imaging time and the number of array elements is shown in Figure 7. N is the number of array elements in both the *x* cross range and *y* cross range. It can be seen that the time cost of the proposed FSA increases slowly with the increase of the number of array elements, while that of RMA increases dramatically. It indicates that the proposed FSA is more suitable for MMW holographic imaging when the number of array elements is large.

### 3.3. Experimental Results

**Figure 8:**
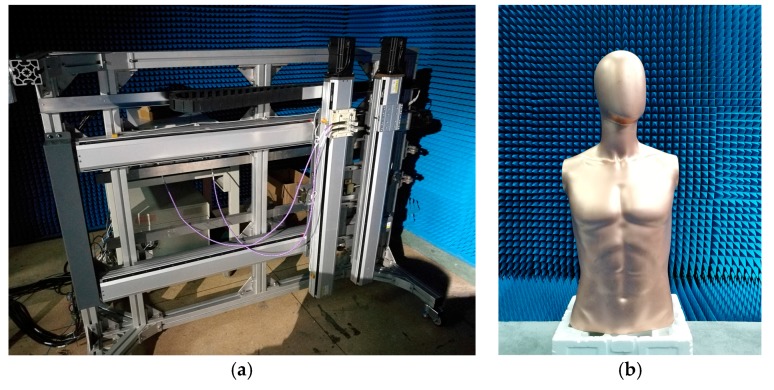
Experimental scene. (**a**) Experimental system; (**b**) Optical image of the mannequin.

In order to validate the behavior of the proposed method in practice, a near-field planar millimeter-wave holographic prototype imager was developed in the 35 GHz band. The experimental system parameters were the same as the point targets simulation condition. There was a mannequin located at a perpendicular distance of $z_{c}$ = 0.43 m away from the measured antenna array. The echo data was collected by a pair of horn antennas scanning over a square aperture of 64 cm × 84 cm with a 4 mm spatial sampling interval in both *x*- and *y*-directions. The two antennas were nearly bound to each other, so it could be regarded as monostatic, and the transmitting antenna was located at the front of the receiving antenna so as to avoid signal coupling. The experimental system is shown in Figure 8a and the mannequin to be imaged is shown in Figure 8b.

**Figure 9:**
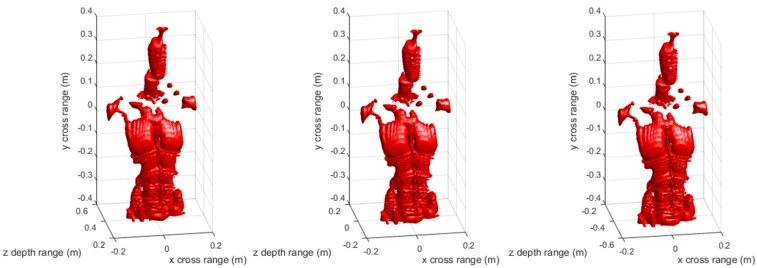
Three-dimentional imaging results of the mannequin by conventional RMA with compensation distance 0 (**left**), 0.43 m (**middle**), and 0.86 m (**right**).

**Figure 10:**
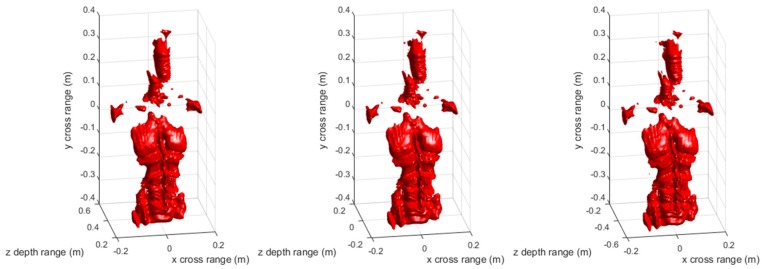
Three-dimensional imaging results of the mannequin by proposed FSA with compensation distance 0 (**left**), 0.43 m (**middle**), and 0.86 m (**right**).

Figure 9 and Figure 10 are the 3-D reconstructed images of the mannequin by compensating different center distances $z_{c}$ of the imaging scene with conventional RMA and proposed FSA, respectively. The dynamic range in both Figure 8 and Figure 9 is −20 ~ 0 dB. Similar to the point targets simulation results, the different compensation distance has almost no effect on 3-D imaging results of the mannequin by RMA and FSA. The experimental results have fully proved that the proposed 3-D FSA has a large depth of focus.

**Figure 11:**
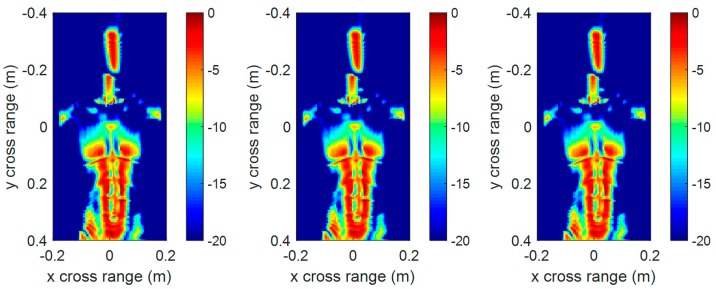
The front view of 3-D imaging results by conventional RMA with compensation distance 0 (**left**), 0.43 m (**middle**), and 0.86 m (**right**).

**Figure 12:**
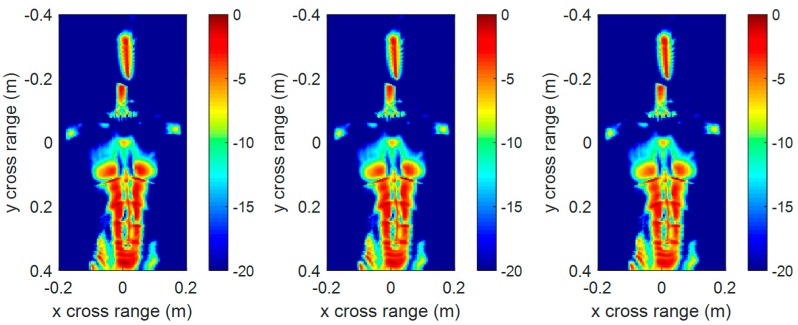
The front view of 3-D imaging results by conventional RMA with compensation distance 0 (**left**), 0.43 m (**middle**), and 0.86 m (**right**).

The 3-D imaging results do not include scattering intensity information. To further compare the imaging quality, Figure 11 and Figure 12 show the front view of the 3-D reconstructed image in Figure 9 and Figure 10, respectively. It can be seen that the front view of the 3-D imaging results between RMA and FSA are comparable, and the compensation distance has almost no effect on the imaging results.

In order to acquire a quantitative analysis of the imaging quality, entropy was introduced to evaluate the focusing quality of the imaging results. Entropy is widely used in the autofocusing techniques of SAR imaging [21,22] to evaluate the focusing quality of an SAR image. The smaller the entropy is, the better the image quality. We calculated the entropy of the above front view images, as given in Table 3. The entropy of the proposed FSA was a little smaller than that of RMA because of the truncation effect of the interpolating kernel function in RMA. On the whole, the imaging performance of the two methods is comparable.

In order to guarantee the imaging quality of RMA, the interpolation interval was chosen as the spatial frequency sampling interval of the transmitted signal, and the interpolation kernel function was the interp1 function in Matlab with a linear mode. The computational time of RMA and FSA were 28.3548 s and 10.8127 s, respectively. Compared with the conventional RMA, the proposed algorithm is more efficient.

## 4. Conclusions

In this paper, a fast 3-D FSA with large depth of focus is presented for near-field planar MMW holographic imaging. The 3-D FSA takes the cross-range range coupling term, which is neglected in conventional RMA, into consideration and performs the range cell migration correction for de-chirped signal without interpolation by using a 3-D frequency scaling operation. The key step of the proposed algorithm is the introduction of a 3-D frequency scaling operator to eliminate the space variation of range cell migration, which improves the focusing depth. Simulation and experimental results have proved that the 3-D FSA proposed in this paper is comparable in accuracy and more efficient when compared with conventional RMA. Our method can be directly used for safety inspection in a near-field planar MMW holographic imaging system, and the performance can be further improved by adopting parallel computation of the graphics processing unit.

## Figures and Tables

**Table 1:** Computational complexity of the 3-D frequency scaling algorithm. RVP = residual video phase.

Operation	Real Multiplications	Real Additions	Sine and Cosine
2-D azimuth FFT	$2N_{x}N_{y}N_{f}(\log_{2}N_{x}+\log_{2}N_{y})$	$3N_{x}N_{y}N_{f}(\log_{2}N_{x}+\log_{2}N_{y})$	
Multiply by $H_{FS}$	$7N_{x}N_{y}N_{f}$	$3N_{x}N_{y}N_{f}$	$2N_{x}N_{y}N_{f}$
RVP correction	$6N_{x}N_{y}N_{f}\log_{2}N_{f}+7N_{x}N_{y}N_{f}$	$9N_{x}N_{y}N_{f}\log_{2}N_{f}+2N_{x}N_{y}N_{f}$	$2N_{x}N_{y}N_{f}$
Multiply by $H_{IFS}$	$8N_{x}N_{y}N_{f}$	$3N_{x}N_{y}N_{f}$	$2N_{x}N_{y}N_{f}$
Multiply by $H_{RMC}$	$6N_{x}N_{y}N_{f}$	$2N_{x}N_{y}N_{f}$	$2N_{x}N_{y}N_{f}$
Range IFFT	$2N_{x}N_{y}N_{f}\log_{2}N_{f}$	$3N_{x}N_{y}N_{f}\log_{2}N_{f}$	
Multiply by $H_{AREF}$	$6N_{x}N_{y}N_{f}$	$2N_{x}N_{y}N_{f}$	$2N_{x}N_{y}N_{f}$
2-D azimuth IFFT	$2N_{x}N_{y}N_{f}(\log_{2}N_{x}+\log_{2}N_{y})$	$3N_{x}N_{y}N_{f}\;(\log_{2}N_{x}+\log_{2}N_{y})$	
**Total**	$4N_{x}N_{y}N_{f}(\log_{2}N_{x}+\log_{2}N_{y})+$ $8N_{x}N_{y}N_{f}\log_{2}N_{f}+$ $34N_{x}N_{y}N_{f}$	$6N_{x}N_{y}N_{f}\;(\log_{2}N_{x}+\log_{2}N_{y})+$ $12N_{x}N_{y}N_{f}\log_{2}N_{f}+$ $12N_{x}N_{y}N_{f}$	$10N_{x}N_{y}N_{f}$

**Table 2:** Coordinates of ideal point targets.

Target Number	*x*-Axis (m)	*y*-Axis (m)	*z*-Axis (m)
1	0	0	0.51
2	−0.1	0	0.51
3	0.1	0	0.51
4	0	−0.1	0.51
5	0	0.1	0.51

**Table 3:** Entropy of the front view images.

Compensation Distance (m)	RMA	FSA
0	8.5002	8.3528
0.43	8.5008	8.3859
0.86	8.5010	8.4195

## Appendix A

In this section, the derivation of Equation (10) is presented. We have

(A1) $$\begin{array}{cc} S\;(K_{x},K_{y},\Delta K_{R}) & =A_{1}\;(K_{x},K_{y},\Delta K_{R})\;\exp\;(-j(\frac{z}{A_{XY}}-R_{ref})\Delta K_{R})\;\exp\;(-jA_{XY}K_{Rc}z-jK_{x}x-jK_{y}y)\\ & \times \exp\;(j\frac{(K_{x}^{2}+K_{y}^{2})z}{2K_{Rc}^{3}A_{XY}^{3}}\Delta K_{R}^{2})\;\exp\;(-j\frac{(K_{x}^{2}+K_{y}^{2})z}{2K_{Rc}^{4}A_{XY}^{5}}\Delta K_{R}^{3})⊗\exp\;(-j\frac{\Delta K_{R}^{2}}{2b}) \end{array}$$

(A2) $$H_{FS}(K_{x},K_{y},\Delta K_{R})=\exp\;(-j\frac{\Delta K_{R}^{2}}{2b}(A_{XY}-1))$$

Multiplying Equation (A1) by Equation (A2), the convolution integral of the product is

(A3) $$\begin{array}{cc} S_{1}(K_{x},K_{y},\Delta K_{R}) & =\int A_{1}(K_{x},K_{y},\Delta L)\;\exp\;(-j(\frac{z}{A_{XY}}-R_{ref})\Delta L)\;\exp\;(-jA_{XY}K_{Rc}z-jK_{x}x-jK_{y}y)\\ & \times \exp\;(j\frac{(K_{x}^{2}+K_{y}^{2})z}{2K_{Rc}^{3}A_{XY}^{3}}\Delta L^{2})\exp\;(-j\frac{(K_{x}^{2}+K_{y}^{2})z}{2K_{Rc}^{4}A_{XY}^{5}}\Delta L^{3})\\ & \times \exp\;(-j\frac{\Delta K_{R}^{2}}{2b}(A_{XY}-1))\exp\;(-j\frac{{\left(\Delta K_{R}-\Delta L\right)}^{2}}{2b})d\Delta L \end{array}$$

The last two index terms in Equation (A3) can be transformed as

(A4) $$\begin{array}{ll} & \exp\;(-j\frac{\Delta K_{R}^{2}}{2b}(A_{XY}-1))\exp\;(-j\frac{{\left(\Delta K_{R}-\Delta L\right)}^{2}}{2b})\\ = & \exp\;(-j\frac{A_{XY}}{2b}{\left(\Delta K_{R}-\frac{\Delta L}{A_{XY}}\right)}^{2})\exp\;(j\frac{(1-A_{XY})\Delta L^{2}}{2bA_{XY}}) \end{array}$$

Substituting Equation (A4) into Equation (A3), we have

(A5) $$\begin{array}{cc} S_{1}(K_{x},K_{y},\Delta K_{R}) & =\int A_{1}(K_{x},K_{y},\Delta L)\;\exp\;(-j(\frac{z}{A_{XY}}-R_{ref})\Delta L)\;\exp\;(-jA_{XY}K_{Rc}z-jK_{x}x-jK_{y}y)\\ & \times \exp\;(j\frac{(K_{x}^{2}+K_{y}^{2})z}{2K_{Rc}^{3}A_{XY}^{3}}\Delta L^{2})\;\exp\;(-j\frac{(K_{x}^{2}+K_{y}^{2})z}{2K_{Rc}^{4}A_{XY}^{5}}\Delta L^{3})\\ & \times \exp\;(-j\frac{A_{XY}}{2b}{\left(\Delta K_{R}-\frac{\Delta L}{A_{XY}}\right)}^{2})\;\exp\;(j\frac{(1-A_{XY})\Delta L^{2}}{2bA_{XY}})d\Delta L \end{array}$$

Letting $\Delta L=A_{XY}\Delta L_{1}$, the Equation (A5) can be transformed as

(A6) $$\begin{array}{cc} S_{1}(K_{x},K_{y},\Delta K_{R}) & =\int A_{1}(K_{x},K_{y},A_{XY}\Delta L_{1})\;\exp\;(-j(\frac{z}{A_{XY}}-R_{ref})A_{XY}\Delta L_{1})\\ & \times \exp\;(-jA_{XY}K_{Rc}z-jK_{x}x-jK_{y}y)\;\exp\;(j\frac{(K_{x}^{2}+K_{y}^{2})z}{2K_{Rc}^{3}A_{XY}^{}}\Delta L_{1}{}^{2})\\ & \times \;\exp\;(-j\frac{(K_{x}^{2}+K_{y}^{2})z}{2K_{Rc}^{4}A_{XY}^{2}}\Delta L_{1}{}^{3})\;\exp\;(-j\frac{A_{XY}}{2b}{\left(\Delta K_{R}-\Delta L_{1}\right)}^{2})\\ & \times \exp\;(j\frac{A_{XY}(1-A_{XY})}{2b}\Delta L_{1}{}^{2})A_{XY}d\Delta L_{1} \end{array}$$

Converting Equation (A6) to convolution form, we finally get

(A7) $$\begin{array}{cc} S_{1}(K_{x},K_{y},\Delta K_{R}) & =A_{2}\exp\;(-j(z-A_{XY}R_{ref})\Delta K_{R})\;\exp\;(-jA_{XY}K_{Rc}z-jK_{x}x-jK_{y}y)\\ & \times \exp\;(j\frac{(K_{x}^{2}+K_{y}^{2})z}{2K_{Rc}^{3}A_{XY}}\Delta K_{R}^{2})\;\exp\;(-j\frac{(K_{x}^{2}+K_{y}^{2})z}{2K_{Rc}^{4}A_{XY}^{2}}\Delta K_{R}^{3})\\ & \times \exp\;(-j\frac{A_{XY}\Delta K_{R}^{2}}{2b}(A_{XY}-1))⊗\exp\;(-j\frac{A_{XY}\Delta K_{R}^{2}}{2b}) \end{array}$$
